# *In Vitro* Bioactivity and Antimicrobial Tuning of Bioactive Glass Nanoparticles Added with Neem (*Azadirachta indica*) Leaf Powder

**DOI:** 10.1155/2014/950691

**Published:** 2014-09-07

**Authors:** M. Prabhu, S. Ruby Priscilla, K. Kavitha, P. Manivasakan, V. Rajendran, P. Kulandaivelu

**Affiliations:** ^1^Centre for Nanoscience and Technology, K. S. Rangasamy College of Technology, Tiruchengode, Namakkal District, Tamil Nadu 637215, India; ^2^Department of Mechanical Engineering, K. S. Rangasamy College of Technology, Tiruchengode, Namakkal District, Tamil Nadu 637215, India

## Abstract

Silica and phosphate based bioactive glass nanoparticles (58SiO_2_-33CaO-9P_2_O_5_) with doping of neem (*Azadirachta indica*) leaf powder and silver nanoparticles were prepared and characterised. Bioactive glass nanoparticles were produced using sol-gel technique. *In vitro* bioactivity of the prepared samples was investigated using simulated body fluid. X-ray diffraction (XRD) pattern of prepared glass particles reveals amorphous phase and spherical morphology with a particle size of less than 50 nm. When compared to neem doped glass, better bioactivity was attained in silver doped glass through formation of hydroxyapatite layer on the surface, which was confirmed through XRD, Fourier transform infrared (FTIR), and scanning electron microscopy (SEM) analysis. However, neem leaf powder doped bioactive glass nanoparticles show good antimicrobial activity against *Staphylococcus aureus* and *Escherichia coli* and less bioactivity compared with silver doped glass particles. In addition, the biocompatibility of the prepared nanocomposites reveals better results for neem doped and silver doped glasses at lower concentration. Therefore, neem doped bioactive glass may act as a potent antimicrobial agent for preventing microbial infection in tissue engineering applications.

## 1. Introduction 

Bioactive glasses allow controlled reactivity and induce a specific biological response that leads to the formation of a biologically active carbonated hydroxyapatite (HAp) layer which is structurally and chemically equivalent to that of the mineral phase of bone [1, 2]. Nanostructured bioactive glasses have gained much attention due to their superior osteoconductive properties when compared with conventional (micron sized) bioactive glass materials. The bioactive glass nanoparticles or nanofibers with polymeric systems enable us to develop potential nanocomposites for orthopedic applications to avoid health risks [3]. Generally, the dissolution rate and microbial infection of implants/biomaterials in orthopedic surgery are still critical health concerns. Infections associated with orthopedic surgery are usually caused by Gram-positive organisms such as* Staphylococcus aureus*,* S. epidermidis*, andstreptococci and Gram-negative organisms such as* Escherichia coli*,* Enterobacter, *and* Pseudomonas aeruginosa* [4].

Recently, bioactive glass nanoparticles doped with antimicrobial agents such as silver, zinc, and magnesium ions have been widely used for clinical applications [5–8]. Similarly, incorporation of metal oxides such as ZnO, MgO, Al_2_O_3_, and TiO_2_ into ceramics and glasses is successfully carried out for tissue engineering purposes [8–11]. Sol-gel derived glass systems possess good textural properties and are capable of accelerating HAp layer formation [12, 13]. Metal oxides are widely used as biomaterials, wherein silver is doped to provide antimicrobial properties against bone infection [14, 15]. Even though silver based glasses release silver ions in a controlled manner for treating bacterial infection, accumulation of silver in bone material may cause metal toxicity to humans. In addition, elevated silver content in bioactive glass may also result in low dissolution rate while replacing calcium ions, poor mechanical property, and* in vitro* behavior [7]. The earlier studies show that the higher silver substitution in bioactive glasses leads to the formation of incipient crystallisation of quartz and, hence, reduces biocompatibility of glass samples [16].

To overcome this obstacle, we have made an attempt to develop bioactive glass nanoparticles by incorporating natural organic substances such as neem, which is an excellent natural antimicrobial agent against broad spectrum of bacteria. Neem (*Azadirachta indica*) is a potent botanical source that has excellent antibacterial, anti-inflammatory, and antiviral properties [17]. The medicinal value of plant is due to the presence of organic/inorganic substances that have a definite physiological action on living organisms. Thus, it is considered as a valuable source of unique natural products for the development of varietal medicines against various diseases and also for the development of industrial products [18]. In this regard, the unique properties of neem have been previously employed for the development of antibacterial polymeric nanocomposite films for food preservative applications [19]. Currently, developing the neem based bioactive glasses and screening for their better* in vitro* bioactivity and cytotoxicity is being an essential task in order to overcome several clinical infections. Hence, bioactive glass nanoparticles doped with neem powder are expected to have better physicochemical properties along with the wide spectrum of antibacterial properties which leads to developing a unique glass composite biomaterial for biomedical applications.

In this study, the aim is to develop a new nanobiomaterial compound from natural sources like neem with good biological activity. In this regard, the nanobioactive glasses added with neem leaf powders are synthesised via simple sol-gel method. The efficiency of neem doped glass nanoparticles is compared with base glass and silver doped nanobioactive glass particles for their physicochemical properties. The bioactivity and antimicrobial activity against* S. aureus* and* E. coli *are explored through systematic studies for tissue engineering applications.

## 2. Material and Methods

### 2.1. Materials

The bioactive glass (SiO_2_-CaO-P_2_O_5_) nanoparticles with 1 mol% of silver and neem leaf powder were prepared by sol-gel method [5, 6]. Tetraethyl orthosilicate (TEOS; 99%; Sigma-Aldrich, India), triethyl phosphate (TEP; 99.5%; HiMedia, India), calcium nitrate (Ca(NO_3_)_2_ *·*4H_2_O; 98%; Merck, India), silver nitrate (99%; Merck), 2 N nitric acid (69%; Merck), ethanol, 1 M ammonia (25%; Merck, India), ultrapure water (Arium 611UF; Sartorius AG, India), and neem leaf powder were used for the preparation of bioactive glass nanoparticles.

### 2.2. Preparation of Neem Leaf Powder

Fresh neem leaves were collected from the Tiruchengode region (Tamil Nadu, India). These leaves were washed several times by double distilled water to remove dust and other impurities and then were shade-dried without exposure to sunlight. The dried leaves were made to ultrafine particles through planetary ball mill (PM100; Retsch, Haan, Germany) in a dry medium at 500 rpm for 3 h. Milling was performed in a 250 mL zirconia grinding jar with 10 mm zirconia balls that were used for millings; ball/charge ratio was 20 : 1. After grinding, the fine particles were collected and stored under nitrogen atmosphere to prevent agglomeration. Finally, these fine particles were used to synthesize neem doped bioactive glass nanoparticles.

### 2.3. Synthesis of Bioactive Glass Nanoparticles

To prepare bioactive glass nanoparticles, initially, TEOS was dissolved in 1 : 1 ratio of ethanol and distilled water and then 2 N nitric acid was added. The solution was then stirred at room temperature for 30 min. After the complete hydrolysis of TEOS, TEP was dropwise added to the stirred precursor and stirring continued for 30 min. Calcium nitrate was dissolved separately with 2 mL distilled water and this solution was added to silica solution at 30 min interval under constant stirring at room temperature. After obtaining the clear solution, 1 M ammonia solution was added drop by drop until the formation of gel, that is, until the solution reaches the pH 8.0. The resulting gel was kept in a hot-air oven at 60°C for 48 h and further dried at 120°C for 48 h. The obtained powder was ground and calcined at 500°C for 4 h to remove the carbon and nitrate impurities. Similarly, silver doped glass was produced by repeating the same procedure followed by addition of silver nitrate solution before the addition of 1 M ammonia solution. The prepared nanobioactive glass samples are hereafter termed as NBG (base glass) and as SNBG (silver doped bioactive glass). The prepared bioactive glass nanoparticles and 1 g fine neem leaf particles were uniformly mixed by planetary ball mill for 15 min. Then, the collected samples were stabilised at 60°C for 2 days to remove the moisture and agglomeration. The prepared neem leaf particles doped bioactive glasses nanoparticles are hereafter termed as NNBG.

### 2.4. Characterisation

The phase analyses of bioactive glass nanoparticles were obtained through X-ray diffraction (XRD) studies using an X-ray diffractometer (X'PertPRO; PANalytical, the Netherlands) with CuK*α* as a radiation (*λ* = 1.5418 Å) at 40 kV with a diffraction angle (2*θ*) varying from 10° to 80°. The infrared spectra of prepared glasses were measured using Fourier transform infrared (FTIR) spectrometer (Spectrum 100; PerkinElmer, USA) at room temperature in the wavenumber range from 4000 to 400 cm^−1^. The particle size, shape, and surface morphology of bioactive glass nanoparticles were studied using transmission electron microscopy (TEM; CM 200; Philips, USA) and scanning electron microscopy coupled with an energy-dispersive X-ray analysis (SEM-EDX, JSM 6360; JEOL, Japan). The purity of the prepared bioactive glass nanoparticles was confirmed by X-ray fluorescence spectrometer (XRF; EDX-720; Shimadzu, Japan). The Brunauer-Emmett-Teller (BET) surface area analyser (Autosorb AS-1MP; Quantachrome, USA) was used to determine the specific surface area (SSA). The NBG and SNBG samples were degassed for 2 h at 290°C and then physisorption analysis was performed with N_2_ adsorption and desorption measurements at liquid N_2_ temperature (−196°C). In case of NNBG, the sample was degassed for 2 h at 80°C to remove the moisture content.

### 2.5. *In Vitro* Bioactivity

The simulated body fluid (SBF) was freshly prepared following the standard protocol [20] using analytical grade chemicals (Merck, India) to explore the* in vitro* bioactivity of the glass samples. The pH value of SBF was 7.4, which is equivalent to that of human blood plasma. From the prepared nanobioactive glass powders (NBG, SNBG, and NNBG), 250 mg was made as pellet using hydraulic pellet maker. The pellets were then immersed separately in 50 mL SBF and incubated at 37°C for 21 days. The ionic changes in the SBF were measured regularly using a pH meter (Orion 5-Star; Thermo Scientific, USA) at an interval of 24 h. After 21 days of immersion, the pellets were removed from the SBF, gently washed with distilled water, and then dried in hot-air oven. The weight loss percentage of sample was calculated according to the following equation:

(1) $$\begin{array}{c} \text{Weight}\;\text{loss}\;\left(\%\right)=\frac{W_{0}-W_{t}}{W_{0}}\times 100, \end{array}$$

where *W*_0_ is the initial weight of the sample and *W*_*t*_ is the weight of the sample measured at time *t* after drying. The above-mentioned experimental procedure was repeated for all samples. After completing the SBF studies, all the prepared pellets were dried at 60°C. The characterisation studies, such as XRD, FTIR, and SEM analysis, were carried out for all samples (NBG, SNBG, and NNBG) to reveal the formation of HAp layer on the glass surface.

### 2.6. Antibacterial Activity

The antimicrobial activity of prepared nanobioactive glass particles was tested against clinical pathogens such as* S. aureus* and* E. coli* using Kirby-Bauer disc-diffusion method [21]. Mueller Hinton agar (MHA) medium (HiMedia, India) was prepared and sterilised at 121°C (15 psi). The MHA plates were prepared by pouring 15 mL molten medium onto sterile Petri plates. The plates were allowed to solidify for about 5 min and 0.1% of culture suspension was swabbed uniformly over the agar until it became invisible. NBG, SNBG, and NNBG pellet samples of 10 mm diameter with 2 mm thickness were placed separately on the freshly inoculated culture plates. The culture plates were incubated at 37°C for 24 h. The diameter of inhibition zones formed around glass disk in all bioactive glass samples was measured in millimeter with a transparent ruler.

### 2.7. Biocompatibility Study

The cytotoxic responses of the prepared glass nanocomposites were screened at different concentrations against human gastric adenocarcinoma cell line (AGS). AGS cell line (ATCC-1739) was obtained from the National Centre for Cell Science, Pune, India. The cells were grown and maintained in Dulbecco's modified Eagle's medium (DMEM)/nutrient mixture F-12 HAM (1 : 1) with 2 mM L^−1^ glutamine supplemented with 10% fetal bovine serum, 45 IU mL^−1^ penicillin, and 45 IU mL^−1^ streptomycin. Growth ingredients were also added and incubated in a humidified atmosphere at 37°C in 5% CO_2_. The morphology of AGS cell lines was observed regularly under binocular inverted microscope. After 48 h of incubation, MTT (3-(4,5-dimethylthiazol-2-yl)-2,5-diphenyltetrazolium bromide) assay was performed to evaluate the viability of the nanobioactive glass-treated AGS cells. The percentage of cell viability from triplicates of the nanobioactive glass-treated and nontreated cells was calculated using optical density (OD_590 nm_) as follows:

(2) Cell  viability  %=OD  of  the  nanoparticles  treated  cellsOD  of  the  cells ×100.

## 3. Results and Discussion

**Figure 1:**
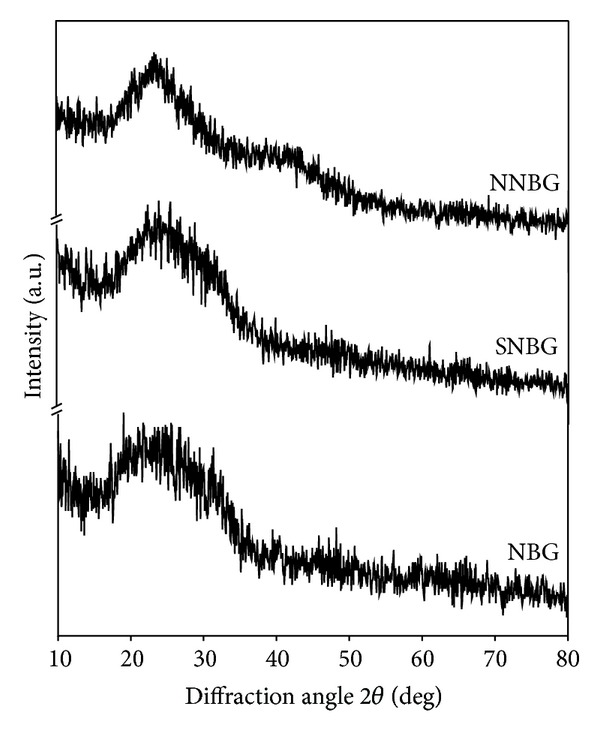
XRD pattern of prepared bioactive glass nanocomposites.

**Figure 2:**
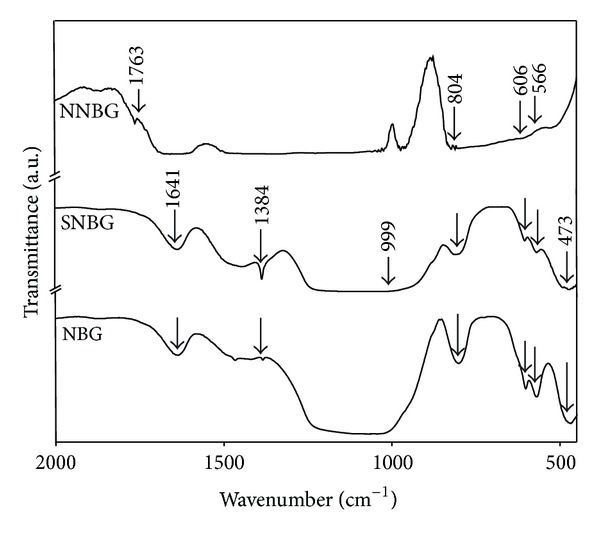
FTIR spectra of bioactive glass nanocomposites.

The XRD pattern of prepared NBG, SNBG, and NNBG is shown in Figure 1. The observed results confirm that there are no diffraction peaks except for broad band observed at 2*θ* values in the range of 20–30°. From the observed results, it is concluded that all glass samples exhibit amorphous nature (JCPDS number 79-1711) without any crystalline peaks and particles reveal that doping of silver and neem leaf powder to the NBG does not influence any changes on its structure of NBG.  Figure 2 shows the FTIR spectra of the synthesised samples (NBG, SNBG, and NNBG). The chemical group, along with the respective frequencies, is given in Table 2. The bands observed at 999, 804, and 473 cm^−1^ correspond to asymmetric and symmetric stretching mode of Si–O–Ca bonds and stretching vibration of Si–O–Si bonds, respectively [22]. A typical absorption band observed at 606 and 566 cm^−1^ corresponds to phosphate (PO_3_^2−^) group [22, 23]. The broad band observed at 1649 and 1384 cm^−1^ corresponds to O–H bending vibration of the chemically adsorbed hydroxyl groups on the glass matrix. Hence, silver (SNBG) and neem (NNBG) doped bioactive glass particles are confirmed by their existing wave number of corresponding functional groups [24]. The presence of terpenoid groups (C=C group and geminal methyl group) in the neem samples 1600 cm^−1^ and 1380 cm^−1^ which are overlapped with the O–H bending vibration [25] confirms the doping of neem with the base glass which are different from other inorganic glass nanocomposites.

**Figure 3:**
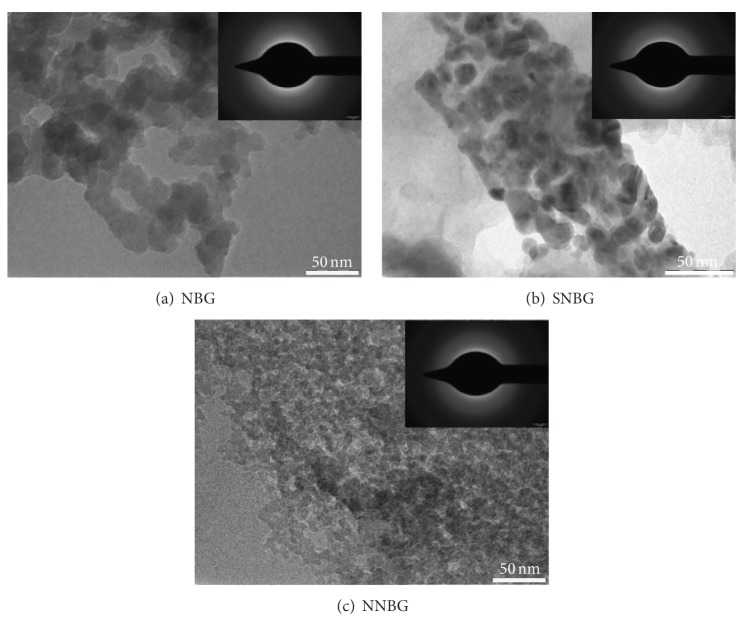
TEM images of prepared bioactive glass nanoparticles.

The size and shape of the prepared bioactive glass nanoparticles are confirmed by TEM analysis (Figure 3). The observed result reveals that the particle size of all glasses is less than 50 nm with uniform spherical morphology. SNBG and NNBG samples show uniform spherical morphology when compared with NBG. Moreover, the selected area electron diffraction pattern (inset in Figure 3) confirms the amorphous nature of the bioactive glass nanoparticles.

**Figure 4:**
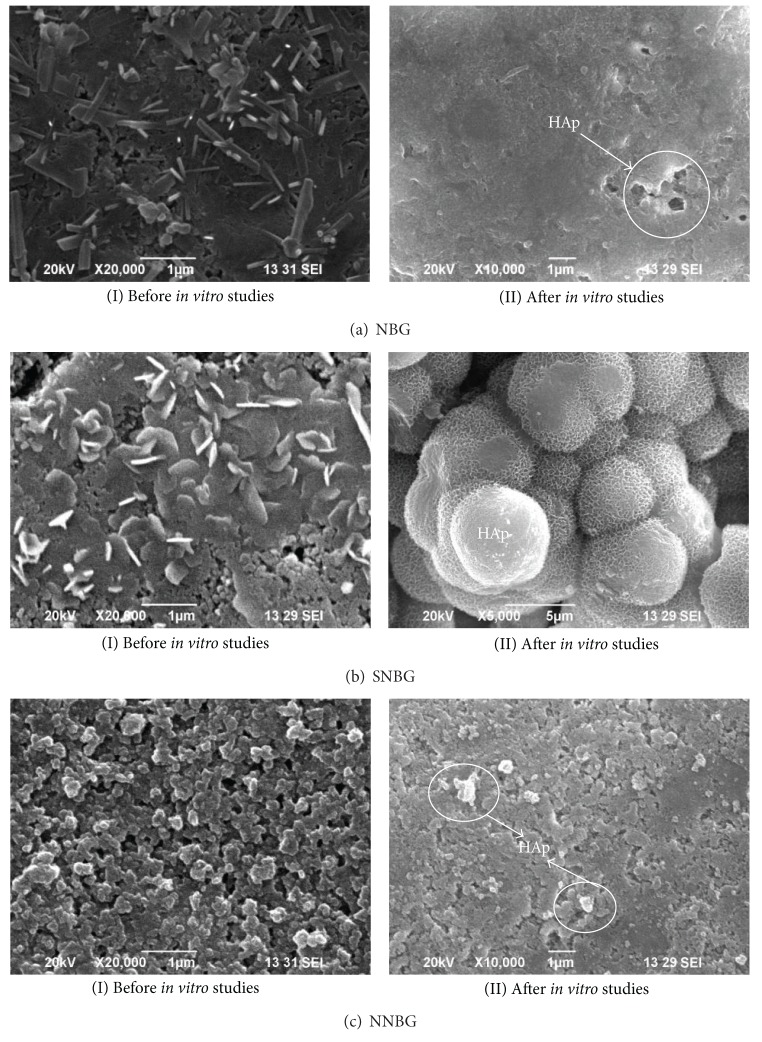
SEM images of silver and neem doped nanobioactive glass particles before and after* in vitro* studies.

The SEM images of the prepared NBG, SNBG, and NNBG samples before* in vitro* studies are shown in Figures 4(a)I, 4(b)I, and 4(c)I, respectively. A bundle of needle shaped surface (Figure 4(a)I) with an irregular morphology is obtained for NBG when compared with SNBG (Figure 4(b)I) and NNBG (Figure 4(c)I) samples. On the other hand, the surface of SNBG and NNBG samples shows, respectively, flake (Figure 4(b)I) and spherical (Figure 4(c)I) morphology. A good spherical morphology with uniform particle size is observed in NNBG samples with slight agglomeration. Moreover, the needle shaped morphology is modified to spherical when the neem particle is doped into glass matrix despite the fact that it does not influence their crystalline structure as observed from XRD pattern. This may be due to the presence of amorphous neem particles and also it reduces the particle size of the glass composite negligibly.  Table 1 summarises the quantitative analysis (wt%) of the prepared bioactive glass nanoparticles, which is obtained from the XRF studies. The percentage of composition of elements present and the weight percentage of the designed and experimentally obtained values are compared. The results show 99% purity with negligible carbon content.

**Figure 5:**
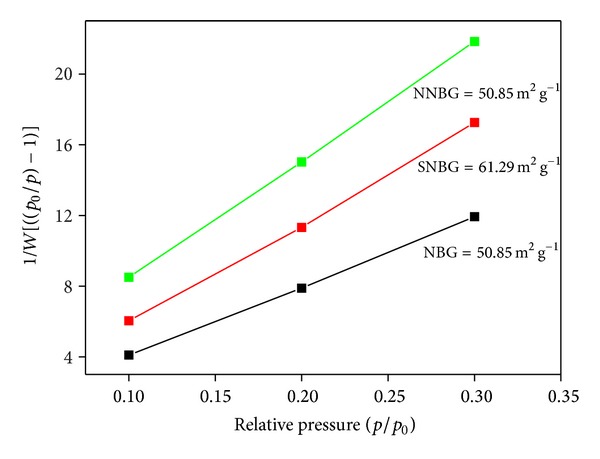
BET plot of prepared bioactive glass nanoparticles.

Figure 5 shows the BET plot of as-synthesised bioactive glass nanoparticles sintered at 500°C. The specific surface areas of NBG, SNBG, and NNBG samples are, respectively, 88.94, 61.29, and 50.85 m^2 ^g^−1^. The addition of neem leaf particles to the NBG reveals lower surface area (50.85 m^2 ^g^−1^) than silver doped nanoparticles (61.29 m^2 ^g^−1^), while comparing the NBG (88.94 m^2 ^g^−1^). The bioactivity of the glass nanoparticles, where the formation of apatite takes place, depends not only on their composition but also on the surface properties [26].

**Figure 6:**
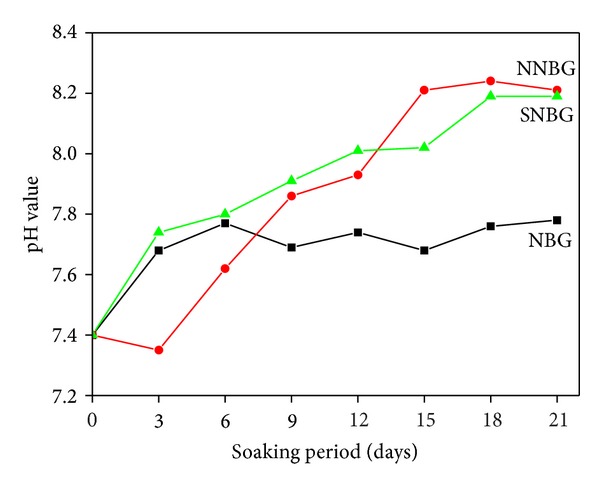
pH value as a function of soaking period in SBF.

The variation in the pH value of the SBF at different immersion intervals for all glass samples is shown in Figure 6. The observed changes in all the samples are due to ion exchange that takes place between the samples and SBF. The NBG and SNBG samples show an increase in the pH value on the third day, whereas NNBG sample reveals a decrease in pH value due to the faster dissolution of hydrophilic starch and cellulose in neem leaf powder. After the third day, the pH value of all samples gradually increases up to the 15th day which may be due to more absorption of supplementary ions and sufficient use of OH^−^ ions from the SBF for HAp layer formation on the glass surface. On the other hand, the deposition of saturated PO_4_^3−^ and Ca^2+^ ions on the glass particles influences the pH value to remain constant after 18th day which is correlated with the previous study [27]. After incubation in SBF, the sample pellets are collected and again characterised using XRD, FTIR, and SEM to ensure the HAp layer formation on the glass surface.

**Figure 7:**
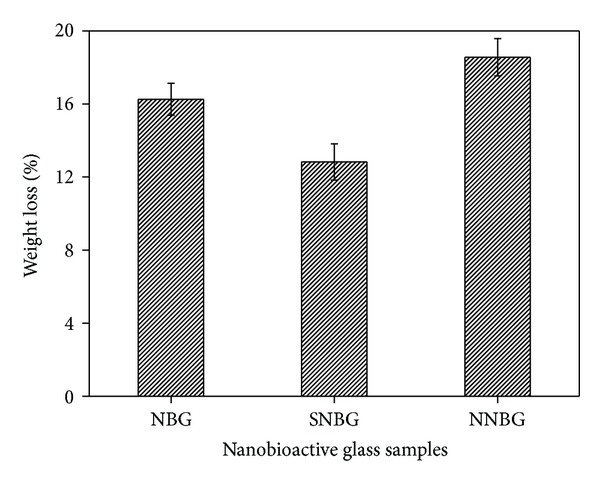
Weight loss percentage of bioactive glass samples after 21 days of incubation in SBF.

The observed weight loss percentage values of NBG, SNBG, and NNBG samples in the SBF are, respectively, 16.26, 12.83, and 18.56% (Figure 7). NNBG shows an increase in weight loss percentage (18.56%) compared with NBG and SNBG, which may be due to hydrophilic nature of carbohydrate content (starch and cellulose, the main constituent in neem leaf) present in the NNBG sample [24]. The observed decrease in weight loss percentage (12.83%) in SNBG compared to NBG is due to substitution of Ag_2_O for CaO, which decreases the degradability of glass and may delay the formation of apatite layer on the glass surface [28].

**Figure 8:**
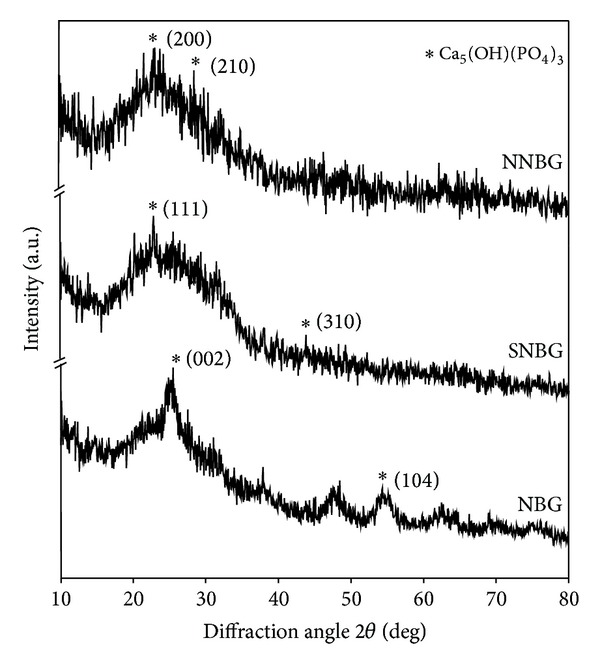
XRD pattern of bioactive glass nanoparticles after* in vitro* studies.

Figure 8 shows the XRD patterns of NBG, SNBG, and NNBG samples after 21 days of* in vitro* studies. The peaks detected at 21.8° (200), 22.9° (111), 25.8° (002), 28.9° (210), 39.8° (310), and 54.4° (104) represent HAp crystalline (JCPDS file number 09-0432) phase over the amorphous glass surface. These results indicate that a strong formation of HAp layer is observed with the NBG and SNBG samples when compared with neem doped glasses. For the NNBG sample, the dissolution rate is suppressed in SBF and acts as an intermediate oxide in the glass samples whereas silver ions influence the low dissolution rate with poor biological properties.

**Figure 9:**
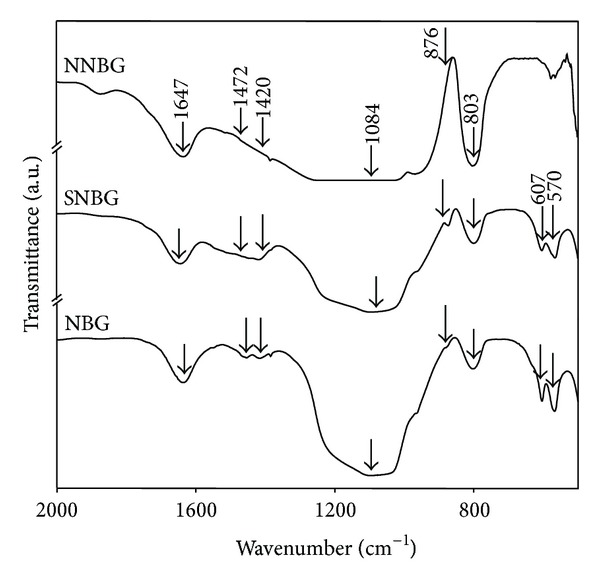
FTIR spectra of bioactive glass nanoparticles after* in vitro* studies.

The observed FTIR spectra of NBG, SNBG, and NNBG samples after immersion in SBF for 21 days are shown in Figure 9 and the peak assignments are given in Table 2. The bands observed at 607 and 570 cm^−1^ correspond to crystalline phase of phosphate (PO_4_^3−^) group [22, 23]. In addition, the peaks observed at 1472, 1420, and 876 cm^−1^ indicate the formation of carbonate apatite [29, 30]. However, it shows that the HAp layer formation is well observed in the SNBG sample when compared with NNBG sample, due to quick dissolution of Ca^2+^ and P^5+^ ions on the surface. This observation shows that SNBG has good bioactivity, as evident from the XRD pattern.

The SEM images of NBG, SNBG, and NNBG samples after 21 days of immersion in SBF are shown in Figures 4(a)II, 4(b)II, and 4(c)II, respectively. The morphological difference is evident in all samples before and after* in vitro* studies (Figure 4). The morphology of NBG appears as light white precipitate because the glass surface is fully reacted in SBF (Figure 4(a)II). Spherical apatite crystals are formed on the surface of the NNBG sample which is confirmed from Figure 4(c)II. The formation of flake like HAp layer is observed on SNBG (Figure 4(b)II), which is in line with the earlier results [31]. These results confirm that the high reactivity of SNBG in SBF shows better* in vitro* bioactivity than the NBG and NNBG samples.

The formation of HAp layer on the surface of glass samples is also revealed through XRF analysis. The observed XRF results for the glass surfaces after immersion in SBF are given in Table 3. The above results confirm that the surface content of Ca^2+^ and P^5+^ increases, whereas the content of Si^4+^ decreases after immersion in SBF for a period of 21 days. After 21 days, the SNBG composite reveals the Ca/P ratio as 1.60, which is in close agreement with the standard stoichiometric value of HAp (1.67). The above result confirms that the formation of HAp layer on the surface of SNBG sample was high when compared with NBG and NNBG samples.

The antimicrobial effect of the NBG, SNBG, and NNBG samples is assessed by the observed zone of inhibition against* S. aureus* and* E. coli*, as given in Table 4. The NBG sample shows no zone of inhibition against both organisms. The zone of inhibition for the NNBG sample is found to be 23 mm against both bacteria. However, the SNBG sample shows the zone of inhibition as 8 and 7 mm against* S. aureus *and* E. coli*, respectively. From the results, it is inferred that neem added glass nanoparticles reveal good antimicrobial activity against Gram-negative and Gram-positive bacteria when compared with silver doped glasses. The observed broad spectrum antibacterial effect exerted by NNBG is superior to SNBG, which is advantageous for clinical applications. Generally, metal/metal oxide doped bioactive glasses are used to confer antimicrobial action for tissue engineering applications [32, 33]. However, in this study, natural source is attempted to replace conventional metal/metal oxide sources as dopant to develop potent antimicrobial agent's doped bioactive glass for clinical applications. The above studies indicate that the SNBG sample shows better* in vitro* bioactivity than the NNBG sample. Thus, using medicinally important botanicals such as neem for the construction of efficient biomaterials, NNBG is less attractive due to its bioactivity.

**Figure 10:**
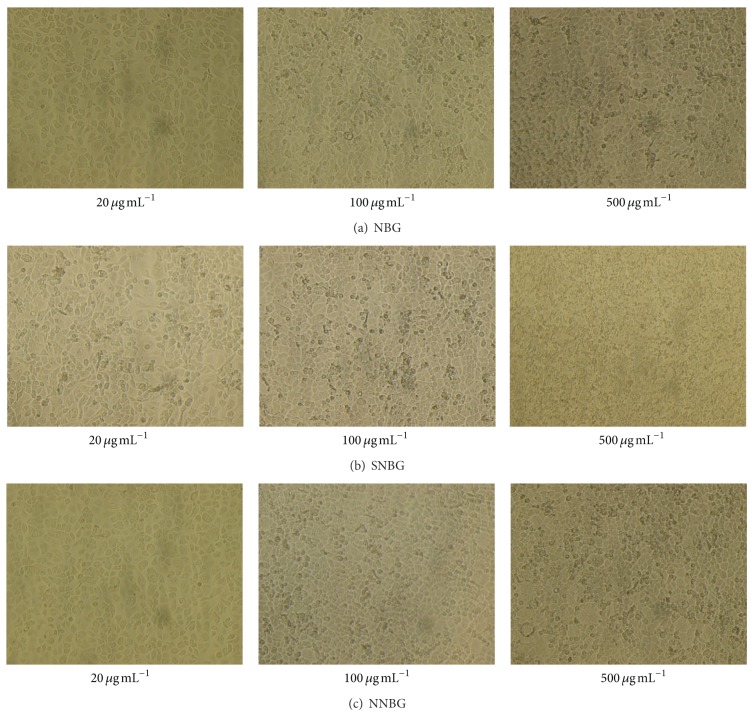
Morphological responses of AGS cell lines exposed to different concentrations of nanocomposites.

**Figure 11:**
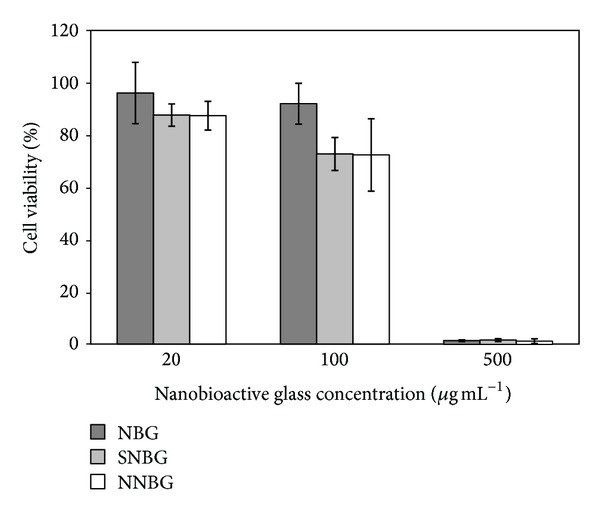
Cell viability percentage of AGS cells treated with nanobioactive glass samples.

Biocompatibility of the prepared nanobioactive glass composites is screened using human adenogastric sarcoma (AGS) cell line. Morphological changes due to the treatment with glass nanocomposites are microscopically observed and are shown in Figure 10. From the result, it is inferred that, as the dosage concentration of nanocomposites in the cell vials increases from 20 to 500 *μ*g mL^−1^, the decrease in cell growth and proliferation are observed by the formation of cell aggregation. Similarly, the cell viability percentage is analysed by MTT assay which exhibits a decrease in cell viability with an increase in glass concentration of all the samples (Figure 11). A decrease in cell viability is found in silver and neem doped glasses when compared with base glass at a concentration of 100 to 500 *μ*g mL^−1^. It is also interesting to note that the* in vitro* biocompatibility of neem doped bioactive glass exhibits enhanced biological responses against AGS cell when compared to silver doped glass samples. The observed results indicate that neem causes only a moderate cell death while doping with base glass samples. This may be due to the fact that neem does not affect the human cells despite the fact that it has potent anticancerous and apoptotic activities. It is also in close agreement with other reported results on the anticancerous activity of the neem against human cell lines [34, 35]. Moreover,* in vitro *antibacterial property of the neem does not get altered while doping with base glass. Thus, neem doped nanobioactive glass samples can be used as a potent biomaterial for tissue engineering applications.

## 4. Conclusion

Bioactive glass nanoparticles in the system of 58SiO_2_-33CaO-9P_2_O_5_ doping with neem (*Azadirachta indica*) leaf powder and silver are obtained by the sol-gel method. The prepared glass samples reveal amorphous phase, spherical morphology with a particle size of less than 50 nm. The SSA was found in the range of 50.85–88.94 m^2 ^g^−1^. The* in vitro* bioactivity and antimicrobial activity for NNBG and SNBG are studied and compared with NBG sample. The results evidenced that the HAp layer formation is more in NBG and SNBG than in NNBG. In contrast, the antimicrobial study reveals that NNBG has good antibacterial activity against* S. aureus* and* E. coli *compared with SNBG. In addition, biocompatibility test against human gastric adenocarcinoma cell line also substantiates the better biocompatibility in NNBG at lower concentration. From the bioactivity and antimicrobial activity results, it is concluded that NNBG could be potential candidate for tissue engineering applications because of its excellent antimicrobial and* in vitro* biocompatibility properties.

## Figures and Tables

**Table 1:** Compositions of prepared bioactive glass nanoparticles through XRF analysis.

Sample code	Designed (wt%)					Experimental (wt%)			
	SiO_2_	CaO	P_2_O_5_	Ag_2_O	Neem powder	SiO_2_	CaO	P_2_O_5_	Ag_2_O
BG	58	33	9	0	0	59.23	36.440	4.33	0
SNBG	58	32	9	1	0	63.58	30.67	4.72	0.97
NNBG	58	32	9	0	1	58.07	36.54	5.31	0

**Table 2:** Analysis of FTIR spectra of the bioactive glass nanoparticles.

Wavenumbers (cm^−1^)						Peak assignments	References
Before *in vitro *			After *in vitro *				
NBG	SNBG	NNBG	NBG	SNBG	NNBG		
473	473	—	—	—	—	Si–O–Si stretching	[22]
566	566	566	570	570	570	PO_3_^−2^ vibration band	[22]
606	606	606	607	607	607	–P=O bending band, PO_4_^−3^ vibration band	[22, 23]
804	804	804	807	807	807	Symmetric Si–O–Si stretching in SiO_4_ tetrahedron	[22]
—	—	—	876	876	876	C–O stretching vibration band in CO_3_^2−^	[28, 29]
999	999	999	1084	1084	1084	Asymmetric Si–O–S stretching in SiO_4_ tetrahedron	[22]
1384	1384	1384	—	—	—	O–H bending vibration band	[22]
—	—	—	1420	1420	1420	C–O stretching vibration band in CO_3_^2−^	[28]
—	—	—	1472	1472	1472	C–O stretching vibration band in CO_3_^2−^	[28]
1641	1641	1641	1647	1647	1647	O–H bending (molecular water)	[22]

**Table 3:** Elemental analysis of NBG, SNBG, and NNBG sample surfaces after immersion in SBF by XRF.

Samples	After immersion in SBF		
	Ca (wt%)	P (wt%)	Ca/P
NBG	38.96	18.55	2.10
SNBG	36.74	22.82	1.60
NNBG	34.28	27.86	1.23

**Table 4:** Antimicrobial effect of bioactive glass nanocomposites against clinical pathogens.

Serial number	Organisms	Zone of inhibition (mm)		
		NBG	SNBG	NNBG
1	*Staphylococcus aureus *	—	8	23
2	*Escherichia coli *	—	7	23
